# Machine Learning Classification for Assessing the Degree of Stenosis and Blood Flow Volume at Arteriovenous Fistulas of Hemodialysis Patients Using a New Photoplethysmography Sensor Device

**DOI:** 10.3390/s19153422

**Published:** 2019-08-04

**Authors:** Pei-Yu Chiang, Paul C. -P. Chao, Tse-Yi Tu, Yung-Hua Kao, Chih-Yu Yang, Der-Cherng Tarng, Chin-Long Wey

**Affiliations:** 1 Institute of Electrical Control Engineering, National Chiao Tung University, Hsinchu 300, Taiwan; 2 Department of Electrical Engineering, National Chiao Tung University, Hsinchu 300, Taiwan; 3 Division of Nephrology in Taipei Veterans General Hospital, Taipei 11217, Taiwan

**Keywords:** photoplethysmography (PPG) sensor, arteriovenous fistula (AVF), hemodialysis (HD) patients, machine learning classifiers, support vector machine (SVM)

## Abstract

The classifier of support vector machine (SVM) learning for assessing the quality of arteriovenous fistulae (AVFs) in hemodialysis (HD) patients using a new photoplethysmography (PPG) sensor device is presented in this work. In clinical practice, there are two important indices for assessing the quality of AVF: the blood flow volume (BFV) and the degree of stenosis (DOS). In hospitals, the BFV and DOS of AVFs are nowadays assessed using an ultrasound Doppler machine, which is bulky, expensive, hard to use, and time consuming. In this study, a newly-developed PPG sensor device was utilized to provide patients and doctors with an inexpensive and small-sized solution for ubiquitous AVF assessment. The readout in this sensor was custom-designed to increase the signal-to-noise ratio (SNR) and reduce the environment interference via maximizing successfully the full dynamic range of measured PPG entering an analog–digital converter (ADC) and effective filtering techniques. With quality PPG measurements obtained, machine learning classifiers including SVM were adopted to assess AVF quality, where the input features are determined based on optical Beer–Lambert’s law and hemodynamic model, to ensure all the necessary features are considered. Finally, the clinical experiment results showed that the proposed PPG sensor device successfully achieved an accuracy of 87.84% based on SVM analysis in assessing DOS at AVF, while an accuracy of 88.61% was achieved for assessing BFV at AVF.

## 1. Introduction

The arteriovenous fistula (AVF), which refers to the surgical connection between an artery and a vein at the forearm, is the lifeline of chronic kidney disease (CKD) patients for performing hemodialysis (HD) treatment. It is well known that after long-term HD treatments, the endothelia cell at the AVF may suffer from various lesions, such as thrombosis, inflammation, hyperplasia or calcification, etc., which may lead to failures of H.

D treatment [1]. According to the National Kidney Foundation [1], AVF can be assessed by evaluating the degree of stenosis (DOS) and blood flow volume (BFV) flowing inside. In hospitals, the DOS at AVF is assessed by a non-invasive Doppler machine or invasive angiography. On the other hand, BFV at AVF is measured also by a non-invasive Doppler machine or invasive concentration dilution methods. However, all conventional methods need well-trained operators as well as expensive and bulky instruments. Therefore, neither of them are possible of becoming popular in the home-care market as small-sized sensors. Toward inexpensive, convenient measurements, there were some published works devoted to developing small-sized sensors for evaluating DOS and/or BVF non-invasively.

As for measuring DOS, different researchers have dedicated efforts to smaller-sized sensors based on acoustic phonocardiograms (PCGs), optical photoplethysmography (PPG), and ultra-sound Doppler. Wang et al. [2] presented a small-sized stethoscope auscultation sensor with a radial basis neural network, which shows an accuracy of 87.84% in detecting the DOS of AVF. Chen et al. [3] published a PCG sensor system for evaluating DOS at AVF using a fuzzy Petri net, which reached a 95% accuracy. Yieh et al. [4] proposed an SVM algorithm using a conventional stethoscope auscultation to assess DOS. Although the acoustic sensors show promising results, the high-sensitivity acoustic sensors used for stethoscope auscultation may easily be influenced by unavoidable environmental sound noise, especially in public spaces such as hospitals [5], significantly limiting usability. On the other hand, Wu et al. [6] presented a bilateral PPG sensor system to evaluate DOS at AVF by a cooperative game algorithm, which results in a correlation greater than 0.9. Du and Stephanus [7,8] published works assessing DOS at AVF using bilateral PPG sensors and achieved 94.82% in accuracy. However, the size of the bilateral PPG sensor was too large to transform into a portable or wearable device for homecare, not to mention only 11 subjects were tested [8]. Wu et al. [9] developed a small-sized ultra-sound Doppler module to assess DOS, which suffers, as opposed to the bulky ultra-sound Doppler machine, from the variations induced by environmental interference, different operators, and techniques of digital post-signal processing. In this work, a single, small-sized, hand-held PPG sensor [10] was utilized for assessing DOS at AVF based on measured PPG waveforms. The sensor system was successfully designed to minimize the environmental lighting interference for high accuracy.

As for measuring BFV non-invasively using small-sized and inexpensive sensors, recently, Webb et al. [11] proposed a patch-type thermal sensor for measuring BFV by sensing the heat transportation of microcirculation. The results showed a correlation of 0.75. Lee et al. [12] published a magneto-plethysmographic sensor for measuring blood flow, resulting in a correlation of 0.9355. However, thermal sensors and magnetic sensors radiate heat and an electromagnetic field to patients, which may lead to high-power consumption and, thus, they are not suitable for development as portable devices for long-term monitoring. Zhu et al. [13] used a digital camera and accompanying digital image processing techniques to extract visual pulsation at a patient’s wrist to assess BFV. The resulting accuracy was only 71%, possibly due to the complexity involved in the digital image processing. Chiang et al. [10] presented a single, newly-developed PPG sensor system to monitor and quantify the BFV in AVF with a resulting correlation of 71.76%. The experimental result showed that overestimations occurred for insufficient BFV, which may result in a very high (>50%) type II error (false-negative rate).

Based on the current medical standards of the National Kidney Foundation (NKF) [1], both DOS and BFV are critical in assessing the functionality of AVF. Thus, this study was devoted to developing new algorithms of machine learning classifiers to successfully determine if both DOS and BFV satisfy the NKF’s standards, after which subsequent medical treatments can be undertaken. In terms of accuracy and ubiquitous usage of the new hand-held PPG module developed, the work presented herein well demonstrates favorable performance as opposed to all the aforementioned prior studies. This work is organized as follow: In Section 2, the theories and principles of the PPG sensor and hemodynamics are proposed. Section 3 describes the PPG sensor system and the assessing algorithms. In Section 4, the clinical experiments and the results are presented. Section 5 concludes the work with a discussion.

## 2. Theories and Principles

Theories and principles assessing DOS and BFV at AVF are reviewed in this section to determine the input features for classifier algorithms to later develop.

### 2.1. Beer–Lambert’s Law

**Figure 1:**
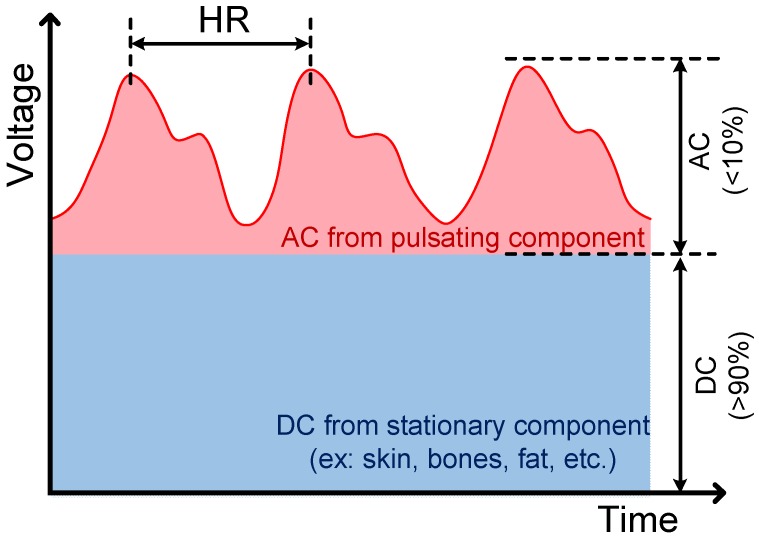
A typical photoplethysmography (PPG) signal, which consists of a very large DC component (>90%) and a very small AC component (<10%) with pulse frequency the same as heart rate (about 50–110 bpm).

Photoplethysmography (PPG) is a non-invasive measuring method aimed at sensing the pulsation of the blood vessel by acquiring the reflective lights from a light-emitted diode (LED) toward a photodiode (PD). Photoplethysmography sensors are known for their non-invasive, small-sized, inexpensive, easy-to-use measurements, and it is widely used in hospitals for monitoring heart rate [14], heart rate variability [15], blood pressure [16], blood oxygen level [17], etc. A typical PPG signal is shown in Figure 1, where the PPG signal is composed of a very large direct component (DC) and a very small alternating component (AC). The DC results from stationary tissues, such as veins, skins, and bones, etc., while the AC from the pulsation of the measured vessel. The mathematical equation of a PPG signal can be described using the known physical theory, Beer–Lambert’s law, as:

(1) $$I_{r}=I_{0}\cdot exp\left(\varepsilon_{t}C_{t}S_{t}\right)\cdot exp\left(\varepsilon_{b}C_{b}S_{b}\right),$$

where *I_r_* denotes the received light intensity from the PD; *I*_0_ denotes the intensity of the incident light; *ε_b_* and *ε_t_* denote the light absorption coefficients of blood and tissues, respectively; *c_b_* and *c_t_* denote the Mohr concentrations of blood and tissues, respectively; *s_b_* and *s_t_* denote the transmission paths of light of blood and tissues, respectively. Note that *s_b_* is time-varying due to the diameter changes of the measured blood vessel. Moreover, by applying the Taylor approximation to the exponential function in Equation (1), one can obtain:

(2) $$\begin{array}{cc} I_{r} & =I_{0}\cdot exp\left(\varepsilon_{t}c_{t}s_{t}\right)\cdot \left(1+\varepsilon_{b}c_{b}s_{b}\right),\\ & =I_{0}\cdot exp\left(\varepsilon_{t}c_{t}s_{t}\right)+I_{0}\cdot exp\left(\varepsilon_{t}c_{t}s_{t}\right)\varepsilon_{b}c_{b}s_{b},\\ & =DC+AC. \end{array}$$

To derive the length of the light transmission path with the aim to reduce the interference from other tissue, the 2nd term in Equation (2), AC, is normalized by DC, yielding:

(3) $$s_{b}=\frac{1}{\varepsilon_{b}c_{b}}\frac{AC}{DC},$$

where the effects of other tissue (*ε_t_*, *c_t_*, and *s_t_*) are normalized. Note that the AC/DC is generally defined as perfusion index (PI) [18,19]. Moreover, considering the blood oxygen level, the term *ε_b_c_b_* in Equation (3) can be expanded to [20]:

(4) $$\varepsilon_{b}c_{b}=SpO_{2}\cdot \varepsilon_{HbO}+\left(1-SpO_{2}\right)\varepsilon_{Hb},$$

where *SpO*_2_ denotes the blood oxygen saturation level; *ε_HbO_* and *ε_Hb_* denote the light absorption coefficients of oxy-hemoglobin and hemoglobin, respectively. Combining Equations (3) and (4) yields:

(5) $$PI\equiv \frac{AC}{DC}=s_{b}\left[SpO_{2}\cdot \varepsilon_{HbO}+\left(1-SpO_{2}\right)\varepsilon_{Hb}\right],$$

where *PI* denotes the perfusion index. In Equation (5), the light transmission path *s_b_* is, in fact, proportional to the time-varying diameter of the measured blood vessel in pulsation. This equation is important for deriving BFV and DOS in the following sections.

### 2.2. Hemodynamic Models

**Figure 2:**
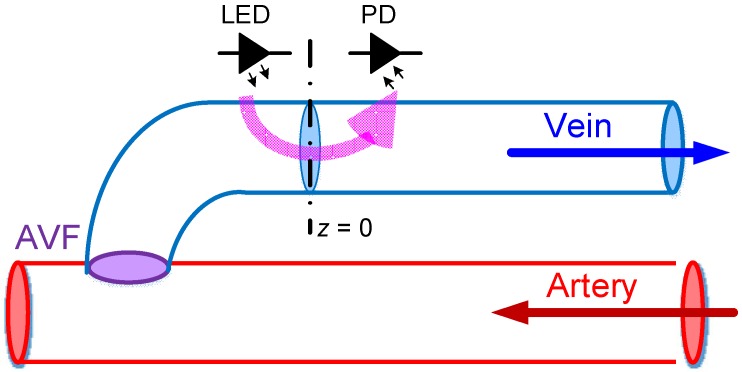
The coordinate axis used in this work, where the *z*-axis lies along the blood vessel with the measuring spot assumed to be at *z* = 0.

To derive the input features for assessing BFV, the hemodynamic model of AVF is derived herein. In this work, the telegrapher equations were introduced to solve the hemodynamic model of AVF. At first, the momentum and mass conservation equations of AVF can be prescribed by [21]:

(6) $$-\frac{\partial p\left(z,\;t\right)}{\partial z}=Rq\left(z,t\right)+L\frac{\partial q\left(z,\;t\right)}{\partial t},$$

(7) $$-\frac{\partial q\left(z,\;t\right)}{\partial z}=Gp\left(z,t\right)+C\frac{\partial p\left(z,\;t\right)}{\partial t},$$

where *q*(*z*,*t*) and *p*(*z*,*t*) denote the instantaneous BFV and blood pressure (BP) at location *z* and at time *t*, respectively; *z* denotes the axis along the AVF, as shown in Figure 2. Also, in Equations (6) and (7),

(8) $$R=c_{1}\frac{128\eta }{d_{0}^{4}};\;L=c_{2}\frac{4\rho }{\pi d_{0}^{2}};\;G=0;\;C=\frac{\left(1-\sigma^{2}\right)\pi d_{0}^{3}}{4hE},$$

*R* denotes the blood vessel resistance; *L* denotes the blood vessel inertance; *G* denotes the influence of vascular branch, which is assumed to be zero; *C* denotes the blood vessel compliance; *η* denotes the dynamic viscosity of blood (about 0.035 g/cm∙s); *ρ* denotes the blood density (about 1.056 g/cm^3^); *σ* denotes the Poisson’s ratio of blood vessel; *d*_0_ denotes the blood vessel diameter at the measuring spot; *h* denotes the thickness of the vessel wall; *E* denotes the Young’s elastic modulus of blood vessel; and *c*_1_ and *c*_2_ are the parameters describing the influence from pulsation of heart rate, which can be approximated by [22]:

(9) $$c_{1}=0.18W+0.45,\;c_{2}=-0.018W+1.39,$$

where *W* denotes the Womersley number. This number is defined as [23]:

(10) $$W=d_{0}\sqrt{\frac{\omega \rho }{4\eta }},$$

where *ω* denotes the heart rate frequency. It is assumed that *R*, *L*, and *C* are independent from *t* and *z*. Also, it is assumed that blood vessels are in finite lengths. Therefore, Equations (6) and (7) can be solved with solutions analogized to the well-known telegrapher equation (see Appendix A for details), yielding:

(11) $$p\left(z,t\right)=P_{0}^{+}e^{-\alpha z}\cos\left(-\beta z+\omega t\right),$$

(12) $$q\left(z,t\right)=Q_{0}^{+}e^{-\alpha z}\cos\left(-\beta z+\omega t\right),$$

where *P*_0_^+^ denotes the BP wave along the positive direction, while *Q*_0_^+^ denotes the BFV along the positive direction. Check Appendix A for the definitions of other parameters in Equations (11) and (12). In fact, Equations (11) and (12) are the solutions of the hemodynamic model’s analogy to the telegrapher equation, and they serve well as the essential equations to decide the input features for assessing DOS and BFV at AVF Section 2.3 and Section 2.4

### 2.3. Degree of Stenosis (DOS)

**Figure 3:**
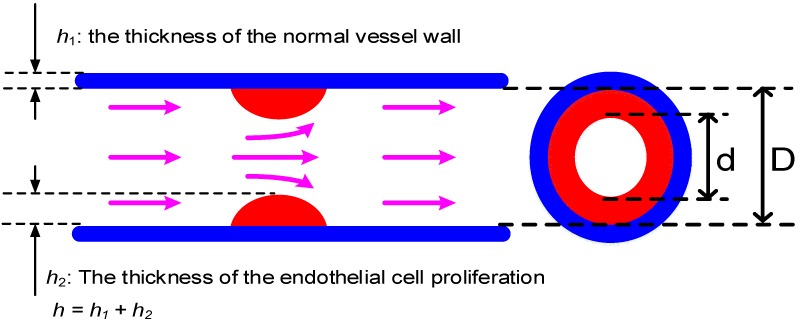
The definition of degree of stenosis (DOS), which is defined as the ratio of the stenosis to non-stenosis areas, where *h*_1_ and *h*_2_ denote the wall thicknesses of normal vessel and the vessel with endothelia cell proliferation.

According to the National Kidney Foundation [1], DOS is defined as the ratio of the cross-sectional area between normal AVF and stenosis AVF, which can be expressed as:

(13) $$DOS=1-\frac{d^{2}}{D^{2}}\times 100\%,$$

where *d* and *D* denote the diameters of the stenosis blood vessel and the normal vessel, respectively, as show in Figure 3. From Figure 3, Equation (13) can be also re-expressed as:

(14) $$DOS=1-\frac{d^{2}}{{\left(d+2h_{2}\right)}^{2}}\times 100\%=1-\frac{d^{2}}{{\left(d+2h-2h_{1}\right)}^{2}}\times 100\%,$$

where *h*_1_ denotes the basic thickness of the blood vessel, which is typically assumed to be a constant; *h*_2_ denotes the thickness of the endothelial hyperplasia. To obtain the vessel thickness *h*, the average blood vessel compliance is introduced [22], which is:

(15) $$C\equiv \frac{A_{max}-A_{min}}{SBP-DBP},$$

where *A_max_* and *A_min_* denote the maximum and minimum cross-sectional areas of the blood vessel; and *SBP* and *DBP* denote the systolic blood pressure (SBP) and diastolic blood pressure (DBP), respectively. Combining Equation (15) and the definition of *C* in Equation (8) leads to a blood-vessel thickness as:

(16) $$h=\frac{\left(1-\sigma^{2}\right)\pi d_{0}^{3}}{E}\frac{SBP-DBP}{d_{max}^{2}-d_{min}^{2}},$$

where *d_max_* and *d_min_* denote the maximum and minimum diameters of the blood vessel, respectively. Actually, in Equation (16), *d_max_* and *d_min_* can be expressed in terms of the length of the light transmission path *s_b_* as seen in Equations (3) and (5), and then substituting Equation (16) into Equation (14) yields:

(17) $$DOS=f_{1}\left(PI_{max},\;PI_{min},\;SpO_{2},\;SBP,DBP\right),$$

where *PI_max_* and *PI_min_* denote the maximum and minimum perfusion indices corresponding to *s_b_*_,*max*_ and *s_b_*_,*min*_, respectively, based on Equation (5). Note further that, based on Equation (5) where AC and DC can be measured by the PPG sensor, *PI_max_* and *PI_min_* in Equation (17) can be obtained. Also, based on the analysis presented in this section, *s_b_*_,*max*_, *s_b_*_,*min*_, and SpO_2_ are all important factors to DOS, and furthermore, they are independent of each other, not only in the mathematical sense, but also physiologically. In short, five independent input features affecting DOS are derived, as shown in Equation (17), to assess DOS at AVF by the classifier algorithms to develop in Section 3.2.

### 2.4. Blood Flow Volume (BFV)

To derive the BFV at AVF, the characteristic impedances of AVF is introduced herein as:

(18) $$Z_{0}\frac{P\left(z\right)}{Q\left(z\right)}=\frac{P_{0}^{+}}{Q_{0}^{+}}=\sqrt{\frac{R+j\omega L}{G+j\omega C}},$$

where *Z*_0_ denotes the characteristic impedances of AVF. Therefore, Equation (12) can be rearranged as:

(19) $$q\left(z,t\right)=\frac{P_{0}^{+}}{Z_{0}}e^{-\alpha z}\cos\left(-\beta z+\omega t\right).$$

Furthermore, it is assumed that the measuring spot of the PPG sensor is placed at *z* = 0, as shown in Figure 2. Hence, Equation (19) becomes:

(20) $$q\left(0,t\right)=P_{0}^{+}\cos\left(\omega t\right)\sqrt{\frac{G+j\omega C}{R+j\omega L}}.$$

To obtain the average BFV, *P*_0_^+^ can be approximated using average mean blood pressure (MBP) as:

(21) $$P_{0}^{+}\equiv MBP=\frac{1}{3}SBP+\frac{2}{3}DBP.$$

The average BFV at AVF can then be obtained by combining Equations (20) and (21), that is:

(22) $$q_{avg}=\left|MBP\sqrt{\frac{G+j\omega C}{R+j\omega L}}\right|,$$

where *q_avg_* denotes the average BFV at AVF, and the absolute symbol is added to obtain the magnitude of complex signals. Combining Equations (8), (15), and (22), all the dependence of the average BFV can be prescribed by:

(23) $$q_{avg}=f_{2}\left(PI_{max},\;PI_{min},\;SpO_{2},\;SBP,DBP,\;\omega \right).$$

As seen in Equation (23), six input features were successfully derived for assessing BFV at AVF via algorithms to develop in Section 3.2.

## 3. Sensor System Design

**Figure 4:**
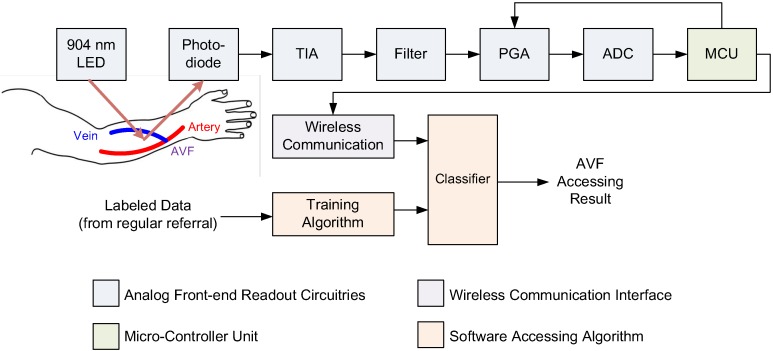
The system architecture of the proposed wireless PPG sensor system for accessing arteriovenous fistulas (AVFs), which is composed of a 904 nm wavelength light-emitting diode (LED), a photodiode (PD), a transimpedance amplifier (TIA), a band-pass filter, an analog–digital converter (ADC), a microcontroller unit (MCU), a wireless communication interface, and the proposed classifiers for accessing DOS and blood flow volume (BFV) of AVFs.

**Figure 5:**
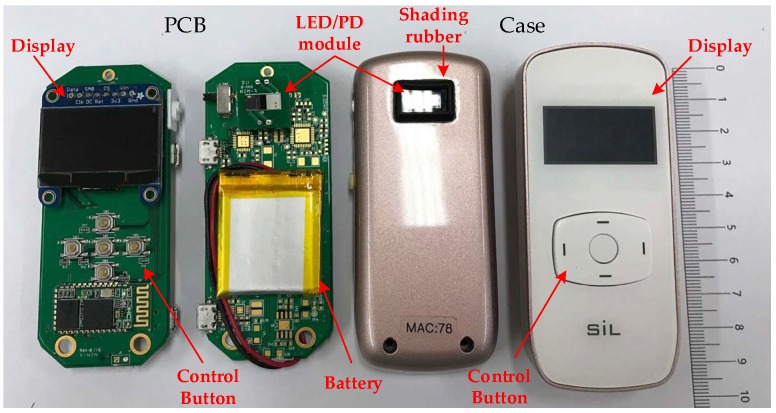
A photo of the proposed portable, wireless, small-sized PPG sensor device for assessing AVF quality.

**Figure 6:**
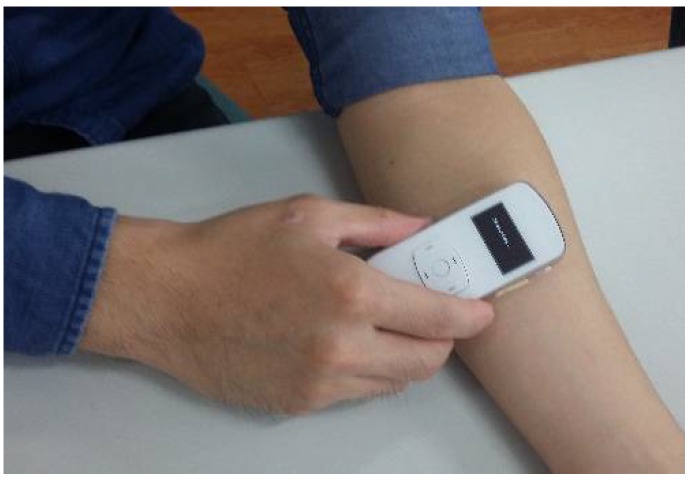
Measurement by the PPG sensor.

With input features determined for classifiers, a readout circuitry was designed and realized, as shown Figure 4. This readout consisted of the front-end analog readout circuitries, micro-controller unit (MCU), wireless communication, and the assessing algorithm. This readout circuitry was implemented on a printed circuit board (PCB). Enclosing the PCB was a newly-developed PPG sensor device, as shown in Figure 5. Also, the photo of measurement using this PPG sensor device is shown in Figure 6. The detailed designs of the circuitry and the algorithms are described in the following.

### 3.1. Readout Circuitry

The analog readout circuitry of the employed PPG sensor device was composed of a light emitted diode (LED), a photodiode (PD), a transimpedance amplifier (TIA), a band-pass filter, a programmable gain amplifier (PGA), and an analog–digital converter (ADC), as shown in Figure 4. First, the light beams emitted from the LED penetrate, refract, and reflect back from the blood vessels in the measured AVF. Then, the intensity changes of the reflected light due to the pulsation of the blood vessels at the AVF are detected and converted by the PD into electrical current signals. Next, a TIA circuit was designed and implemented to transform the current signals from PD into the voltage signals. Third, to deal with motion artifacts and interferences from ambient lighting (50 or 60 Hz), a 4th-order band-pass filter with cut-off frequencies about 0.2 and 10 Hz was proposed to delete the undesired frequency components. Note that the cut-off frequencies are determined in a real-time fashion by the adopted MCU based on the heart rate analyzed from the measured PPG waveforms. This adaptiveness of the cut-offs is highly important, considering the large variations in heart rate from person to person, i.e., approximately 50–110 Hz. Moreover, a tunable PGA circuit was proposed to increase the SNR by controlling the PPG signals to fill the full dynamic range of the ADC. Note that the novel designs of the 4th-order band-pass filter and the tunable PGA make it possible to render PPG waveforms that are almost DC-free and fully dynamic ranged. Finally, the digital PPG signals converted from the ADC are transmitted wirelessly to a smart-phone application or a laptop for the classifier algorithm to assess AVF quality.

### 3.2. Assessing Algorithms

In this work, three different machine learning classifiers are proposed to assess the quality of the AVF, including k-nearest neighbors (kNN), naïve Bayes classifier (NBC), and support vector machine (SVM). The details are described in the followings.

#### 3.2.1. k-Nearest Neighbors (kNN)

The k-nearest neighbors (kNN) is a popular non-parametric supervised learning method used for classification or pattern recognition. It is reported to reveal promising results in PPG sensor applications in biometrics identification [24] and in detecting obstructive sleep apnea [25]. The basic concept of kNN is to classify the testing data by training data with the k nearest Euclidean distance. Although the simple computation makes kNN suitable for implementation in many small-sized devices or into application-specific integrated circuits (ASICs), the computation time may grow without proper feature reduction and/or data compression. Generally, the computation time for the complexity of *N* samples in a D feature dimension using k-nearest samples is in an order of D × *N ×* k. In this work, the distance matrix was calculated using Euclidean distance, while the optimized k value was obtained by a using grid search technique.

#### 3.2.2. Naive Bayes Classifier (NBC)

Naive Bayes classifier is a known supervised learning method for classifying data by applying Bayes theorem under the assumption that all features are independent of each other. Although the independences among all features are always violated in real-world problems, NBC still reveals some robustness in two-class classification due to the fact that the classification results based on the maximum a posterior portability regardless of the probability function of each class. Naive Bayes classifier is widely used in classification applications for PPG sensors such as biometrics [26], cardiac alarming [27], etc. In this work, all feature distributions were assumed to be Gaussian distribution. Therefore, the mathematical equation of the probability density function of the testing data *y* classified to class *C_k_* can be expressed as:

(24) $$p\left(x=y|C_{k}\right)=\frac{1}{\sqrt{2\pi \sigma_{k}^{2}}}exp\left(-\frac{{\left(y-\mu_{k}\right)}^{2}}{2\sigma_{k}^{2}}\right),$$

where *x* denotes a continuous value; *y* denotes the testing data; *C_k_* denotes the *k*th class; *μ_k_* and *σ*^2^*_k_* denote the mean and variance of the training dataset, respectively.

#### 3.2.3. Support Vector Machine (SVM)

The SVM classifier is a popular supervised learning algorithm for data classification, of which the basic concept is to find a hyper-plane separating the two classes with the largest margin. The computation process of SVM can be described as a Lagrange optimization problem in dual quadratic form:

(25) $$L_{p}=\frac{1}{2}\beta^{\prime }\beta +C^{*}\sum_{j}^{n}\zeta_{j}-\sum_{j}^{n}\mu_{j}\zeta_{j}-\sum_{j}^{n}\alpha_{j}\left(y_{i}f^{j}\left(x_{j}\right)-\left(1-\zeta_{j}\right)\right),$$

where *Lp* is the objective function of Lagrange; *f^j^*(*x*) is the separating hyper-plane with pointing vector *β* and bias vector *b*; *α_j_* and *μ_j_* are the Lagrange multiplier; *x_j_* are the features vector with the class label *y_j_*; *ξ_j_* is the slack variable representing the subjects of misclassification; and C* is the penalty for misclassification. Moreover, solving Equation (25) leads to the dual formulation as:

(26) $$\underset{\alpha }{\max}\sum_{j}^{n}\alpha_{j}-\frac{1}{2}\sum_{j}^{n}\sum_{k}^{n}\alpha_{j}\alpha_{k}y_{j}y_{k}x{’}_{j}x_{k},$$

subjected to the constraint:

(27) $$\sum_{j}^{n}y_{j}\alpha_{j}=0,\;0\leq \alpha_{j}\leq C^{*}.\;$$

To deal with non-linear-separable classification problems, the kernel function technique was introduced to transform the dot product *x*’*_j_x_k_* into another features space. In this work, the radial basis function is proposed as the kernel function:

(28) G(xj,xk)=exp(−∥xj−xk∥2σ),

where *G*(*x_j_*,*x_k_*) denotes the radial basis kernel function for substituting the dot product *x’_j_x_k_*; *σ* is a tunable parameter denoting the scaling factors. The optimized value of C* and *σ* are determined by using a grid search technique.

#### 3.2.4. Input Features

According to the discussion in Section 2, the input features for assessing AVF by DOS and BFV are determined based on Equations (17) and (23), respectively, as listed in Table 1. Note herein that the six features selected, as seen in Table 1, are complete based on the thorough physiological analysis leading to Equations (17) and (23) in Section 2.2 and Section 2.3, but not guaranteed independent of each other. This possible non-independence is supposed to be well tackled by the three adopted machine-learning classifiers, especially the SVM that establishes hyperplanes among features to tackle the dependencies. To avoid weighted error, all features are normalized to −1 and 1 before training. Also, to avoid over-fitting results, 10 fold cross validation was introduced in this work. The steps for the 10 fold cross validation process are:

Step (1): randomly divide subjects into 10 subsets.
Step (2): take only one subset for testing and leave the other for training.
Step (3): repeat Step (2) 10 times.
Step (4): calculate the average accuracy and analyze the results.

## 4. Clinical Validation

### 4.1. Experiment Setup

According to the National Kidney Foundation [1], AVFs with DOS larger than 30% are regarded as high-risk in patients. Patients were labeled into DOS-positive and DOS-negative classes. The ground truths of DOS were measured by an ultrasound Doppler machine, Phillips ClearVue 550. In this work, there were a total of 74 subjects who participated in the DOS assessment experiments, including 45 patients labeled as DOS-positive class (DOS < 30%), while 29 patients were labeled as DOS-negative class (DOS > 30%). On the other hand, also according to the National Kidney Foundation [1], the BFV at AVF should be at least 600 mL/min for functional HD treatments. The ground truths of BFV were also obtained by the aforementioned ultrasound Doppler machine, Phillips ClearVue 550. There were a total of 79 subjects who participated in the BFV assessment experiment, including 61 subjects labeled as BFV-positive class (BFV > 600 mL/min), while 18 subjects were labeled as BFV-negative class (BFV < 600 mL/min).

Prior to measurements, all subjects were asked to rest for at least 10 min. During the experiments, the DOS and BFV of each patient were measured and labeled as positive or negative by professional nephrologists. Systolic and diastolic blood pressures were measured with an electronic sphygmomanometer, while the oxyhemoglobin saturation for pulse oximetry (SpO_2_) was measured by a certified oximeter. The proposed PPG sensor was placed at the same measuring spot as the ultrasound Doppler machine. The values of *PI_max_*, *PI_min_*, and HR (ω) were obtained via calculations based on the measured PPG signals. A photo of the proposed PPG sensor device during measurement is shown in Figure 6.

### 4.2. Experiment Results

**Figure 7:**
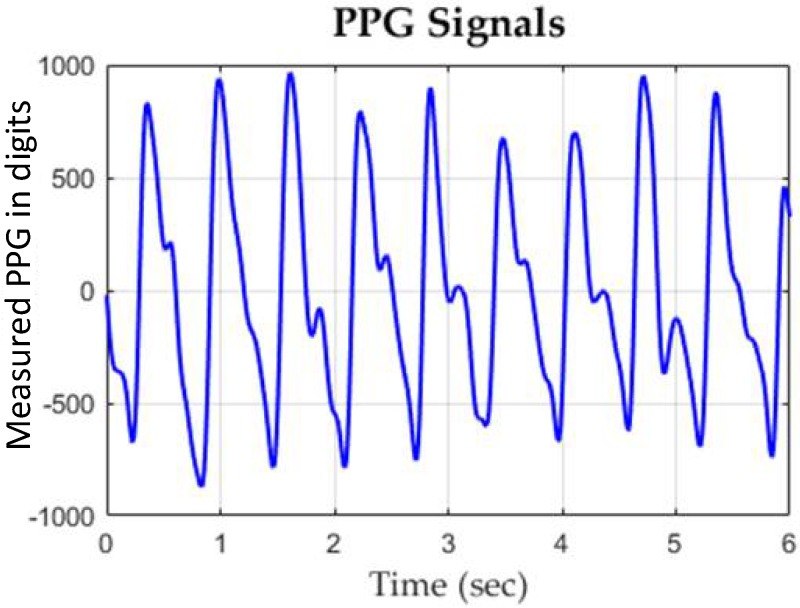
A typical experimental PPG waveform measured by the proposed PPG sensor.

A typical PPG waveform measured by the employed PPG sensor device is shown in Figure 7. The confusion matrix of the classification results of assessing DOS and BFV are shown in Table 2 and Table 3, respectively, where it can be seen that the SVM classifier showed higher accuracies and lower type II error as compared to other methods. A comparison table to other works is given as Table 4.

Observing Table 4, it is clear that the employed PPG sensor device with the designed SVM classifier demonstrated significantly better performance as compared to acoustic sensors in accuracy and type II error for assessing DOS. Although the bilateral PPG sensor [8] shows the highest accuracy in assessing DOS, its sensor’ size are too large to be implemented with a portable or wearable device, not to mention only 11 subjects were tested. On the other hand, for assessing BFV, the proposed PPG sensor device was much better than the authors’ previous work [10] in type II error. Lastly, the employed PPG sensor device with the designed SVM classifier showed better performance compared to the camera sensor [13] in both the sensor’s size and assessing results.

## 5. Conclusions

A newly developed PPG sensor device with a SVM classifier designed for assessing DOS and BFV at AVF were proposed in this work. The optical theory of PPG and the hemodynamic models of AVF were reviewed and solved by an analogy to the telegrapher equation to determine all the possible input features of classifiers for assessing BFV. In addition, the readout circuitry for the PPG sensor device was custom-designed successfully to reduce ambient interference and also improve signal quality. Three classifiers of kNN, NBC, and SVM were applied for assessing AVF. Experiments were conducted with the results showing that the SVM rendered the best performance, successfully achieving the accuracies of 87.84% and 88.61% in assessing AVF quality by DOS and BFV, respectively. These well-demonstrated performances are favorable to the results by all prior arts. The satisfactory accuracies of sensing DOS/BFV presented by the PPG sensor device and the SVM algorithm inside offer hemodialysis patients a convenient device to monitor the quality of their AVFs ubiquitously, including at home. With high accuracies achieved on DOS and BFV individually, future efforts are being undertaken to develop a new classifier to ensure both DOS and BFV are within secure ranges (DOS < 30% and BFV > 600 mL/min) defined by the National Kidney Foundation.

## Figures and Tables

**Table 1:** The input features for assessing DOS and BFV.

Symbol	Features for Assessing DOS	Features for Assessing BFV	Measurement	Description
*The max of peak to peak interval (PI_max_)*	O	O	The perfusion index (AC/DC) by the PPG sensors	The normalized maximum length of light transmission path in blood
*The min of peak to peak interval (PI_min_)*	O	O	The perfusion index (AC/DC) by the PPG sensors	The normalized minimum length of light transmission path in blood
*sphygmomanometer. Oxyhemoglobin saturation by pulse oximetry (SpO*_2_ *)*	O	O	Oximeter	The blood oxygen saturation level
*Systolic Blood Pressure (SBP* *)*	O	O	Electronic sphygmomanometer	The systolic blood pressure
*Diastolic Blood Pressure (DBP* *)*	O	O	Electronic sphygmomanometer	The diastolic blood pressure
*Heart rate (ω)*	X	O	The proposed PPG sensors	The heart rate

**Table 2:** The confusion matrix for assessing DOS.

Samples Number = 74		Ground Truth (Actual Class)	
		Positive	Negative
Classification Results of k-Nearest Neighbors (kNN) (Predicted Class)	Positive	TP = 32 (43.24%)	FP = 5 (6.76%)
	Negative	FN = 13 (17.57%)	TN = 24 (32.43%)
Classification Results of Naive Bayes Classifier (NBC) (Predicted Class)	Positive	TP = 41 (55.41%)	FP = 8 (10.81%)
	Negative	FN = 4 (5.41%)	TN = 21 (28.38%)
Classification Results of Support Vector Machine (SVM) (Predicted Class)	Positive	TP = 42 (56.76%)	FP = 6 (8.11%)
	Negative	FN = 3 (4.05%)	TN = 23 (31.08%)

**Table 3:** The confusion matrix of assessing BFV.

Samples Number = 79		Ground Truth (Actual Class)	
		Positive	Negative
Classification Results of kNN (Predicted Class)	Positive	TP = 51 (64.56%)	FP = 6 (7.59%)
	Negative	FN = 10 (12.66%)	TN = 12 (15.19%)
Classification Results of NBC (Predicted Class)	Positive	TP = 59 (74.68%)	FP = 8 (10.13%)
	Negative	FN = 2 (2.53%%)	TN = 10 (12.66%)
Classification Results of SVM (Predicted Class)	Positive	TP = 59 (74.68%)	FP = 7 (8.86%)
	Negative	FN = 2 (2.53%)	TN = 11 (13.92%)

**Table 4:** Performance comparison table to other prior works.

	H. Y. Wang et al. (2014) [2]	Du Y.-C. et al. (2018) [8]	D. F. Yeih et al. (2014) [4]	J. X. Wu et al. (2015) [9]	P. Y. Chiang et al. (2017) [10]	F. Zhu et al. (2016) [13]	This Work
Sensor	Stethoscope Auscultation	Bilateral PPG	Stethoscope Auscultation	Ultrasound	Single PPG	Camera	Single PPG
Assessing Index	DOS	DOS	DOS	DOS	BFV	BFV	DOS and BFV
Principle	Acoustic	Optical	Acoustic	Doppler	Optical	Optical	Optical
Communication	Wireless	Wired	Wireless	Wired	Wireless	Wired	Wireless
Assessing Algorithm	Neural Network	Neural Network	Support Vector Machine	Color Relation Analysis	Neural Network	Optic Flow Methods	Support Vector Machine
Size	9 cm × 4 cm × 2 cm	Large	-	Large	9 cm × 8 cm × 4 cm	Large	9 cm × 3.5 cm × 1.5 cm
Number of Subjects	479	11	22	50	40	40	DOS: 74 BFV: 79
Accuracy	87.8%	94.82%	84.3%	83%	*R*^2^ = 0.7176 *	R^2^ = 0.71 * (with 32.5% outlier subjects)	DOS: 87.84% BFV: 88.61%
Type II Error	10.75%	-	16.7%	-	>50%	-	DOS: 6.67% BFV: 3.28%

* Correlation to ground truth is regarded as accuracies.

## Appendix A

At first, it is supposed that:

(A1) $$p\left(z,t\right)=P\left(z\right)e^{j\omega t},$$

(A2) $$q\left(z,t\right)=Q\left(z\right)e^{j\omega t},$$

where *P*(*z*) and *Q*(*z*) denote the phasers of *p*(*z*,*t*) and *q*(*z*,*t*), respectively. Substituting Equations (A1) and (A2) into Equations (6) and (7) gives:

(A3) $$\frac{-\partial P\left(z\right)}{\partial z}=\left(R+j\omega L\right)Q\left(z\right),$$

(A4) $$\frac{-\partial Q\left(z\right)}{\partial z}=\left(G+j\omega C\right)P\left(z\right).$$

Taking partial derivatives of Equations (A3) and (A4) with respect to *z* yields:

(A5) $$\frac{-\partial^{2}P\left(z\right)}{\partial z^{2}}=\left(R+j\omega L\right)\frac{\partial Q\left(z\right)}{\partial z},$$

(A6) $$\frac{-\partial^{2}Q\left(z\right)}{\partial z^{2}}=\left(G+j\omega C\right)\frac{\partial P\left(z\right)}{\partial z}.$$

Combining Equations (A3)–(A6) leads to:

(A7) $$\frac{-\partial^{2}P\left(z\right)}{\partial z^{2}}=\left(R+j\omega L\right)\left(G+j\omega C\right)P\left(z\right),$$

(A8) $$\frac{-\partial^{2}Q\left(z\right)}{\partial z^{2}}=\left(R+j\omega L\right)\left(G+j\omega C\right)Q\left(z\right),$$

where the impedance term can be expressed as:

(A9) $$\gamma^{2}=\left(R+j\omega L\right)\left(G+j\omega C\right).$$

Therefore, Equations (A7) and (A8) can be rewritten as:

(A10) $$\frac{-\partial^{2}P\left(z\right)}{\partial z^{2}}=\gamma^{2}P\left(z\right),$$

(A11) $$\frac{-\partial^{2}Q\left(z\right)}{\partial z^{2}}=\gamma^{2}Q\left(z\right).$$

Equations (A10) and (A11) are to be solved for *P* and *Q* in homogenous formats as:

(A12) $$P\left(z\right)=P_{0}^{+}e^{-\gamma z}+P_{0}^{-}e^{\gamma z},$$

(A13) $$Q\left(z\right)=Q_{0}^{+}e^{-\gamma z}+Q_{0}^{-}e^{\gamma z},$$

where *P*_0_^+^ and *P*_0_^−^ denote the BP wave along positive and negative directions, respectively, while *Q*_0_^+^ and *Q*_0_^−^ denote the BFV along positive and negative directions, respectively. The pulse waves from the negative direction, *P*_0_^−^ and *Q*_0_^−^, are the waves reflected from peripheral microvessels. Moreover, it is known that wave velocity of blood (about 10 m/s) is much faster than fluid velocity of blood (about 50 cm/s), thus, the waveforms of *P* and *Q* are superimposed at AVF. Furthermore, considering the amplitude of the reflected wave is small enough at AVF to be considered as negligible as compared to the forward wave, Equations (A12) and (A13) can be approximated by:

(A14) $$P\left(z\right)=P_{0}^{+}e^{-\gamma z},$$

(A15) $$Q\left(z\right)=Q_{0}^{+}e^{-\gamma z}.$$

Substitution of Equations (A14) and (A15) into Equations (A1) and (A2) gives:

(A16) $$p\left(z,t\right)=P_{0}^{+}e^{-\gamma z}e^{j\omega t},$$

(A17) $$q\left(z,t\right)=Q_{0}^{+}e^{-\gamma z}e^{j\omega t},$$

Considering only the real parts of BP and BFV waves give the solutions of BP and BFV as

(A18) p(z,t)=Re⌊P0+e−γzejωt⌋=P0+e−αzcos(−βz+ωt),

(A19) q(z,t)=Re⌊Q0+e−γzejωt⌋=Q0+e−αzcos(−βz+ωt),

where Re denotes the real parts of complex numbers.
